# Advancing Land Monitoring Through Synergistic Harmonization of Optical, Radar and Lidar Satellite Technologies

**DOI:** 10.3390/s25195980

**Published:** 2025-09-26

**Authors:** Ram C. Sharma

**Affiliations:** Department of Informatics, Tokyo University of Information Sciences, 4-1 Onaridai, Wakaba-ku, Chiba 265-8501, Japan; ramc-sharma@digital-ecosphere.org

This Special Issue, entitled “Advancing Land Monitoring through Synergistic Harmonization of Optical, Radar, and Lidar Satellite Technologies,” was launched to link modern sensing with real-world decisions by advancing trustworthy, scalable, and multimodal methods that fuse physics, machine learning, and domain knowledge. With various methods converging in capability, including lidar (GEDI), SAR/optical constellations, UAV platforms, dense in situ networks, and AI accelerators, conditions are ideal to progress from single-sensor case studies to fusion, interpretability, and operational readiness with regard to ecosystems, hazards, cities, and radar systems. The representative contributions to this Special Issue encompass topics such as structural–spectral fusion for wetland and biodiversity mapping; space–air–ground deformation monitoring; radar science spanning bibliometrics, waveforms, and nowcasting; and privacy-preserving urban analytics. Each of these papers push toward actionable insights and proofs of concept. Taking account of the 20 published papers, this collection has been organized into five interrelated, technically coherent themes:

- Registration and alignment foundations: Robust cross-sensor/geometry registration—e.g., fusing displacement fields and emphasizing edges—underpins change detection and any downstream multi-temporal fusion. Several papers prioritize algorithms, datasets, and workflows over socio-economic evaluation, and many focus on single regions/seasons with limited cross-site transfer—gaps the studies themselves acknowledge. This infrastructure enables the exploration of the remaining themes. Accurate alignment makes fusion reliable; without it, uncertainty grows.
- Earth observation for ecosystems: Wetland mapping with GEDI-derived structures, UAV hyperspectral species mapping, and diversity models that pair structure with spectra show that integrating canopy structure (GEDI) with optical/SAR and hydro-topographic context sharpens wetland classes and species maps—reducing spectral vagueness. These advances benefit biodiversity and restoration workflows and motivate uncertainty-aware, open benchmarks.
- Geohazards and land changes: An end-to-end framework fusing SBAS-InSAR, UAV LiDAR, and bathymetry delivers subsidence depth maps and governance-ready accounts—an operational prototype for managing provincial mining impacts. The methods can be generalized in spirit into landslide detection networks that explicitly inject terrain into CNNs, or to robust image registration across viewing geometries, reflecting a recurring topic: physics-informed fusion to curb false positives.
- Radar science and forecasting: A century-scale bibliometric map collects collaboration hubs and emerging AI fronts in weather-radar research, while methodological contributions advance probabilistic diffusion-based nowcasting and optimization-theoretic waveform design. Together, these methods demonstrate the importance of community benchmarks, fair data/format standards, and physics-aware learning—bridges from detection to deployment.
- Urban sensing and public services: Interpretable urban heat mapping (e.g., SHAP-explained drivers), package-level benchmarks for low-cost PM calibration, and federated, pruned models for lightweight traffic forecasting are demonstrated in agency use cases: fast, transparent models that operate under bandwidth and privacy constraints.

Methodologically, this collection advances object-based UAV hyperspectral mapping with genetic-algorithm feature selection; random-forest interpolation of GEDI footprints to 10 m canopy height with demonstrated class-discrimination value; an SBAS-InSAR–UAV LiDAR–bathymetry pipeline achieving ~2 m subsidence depth; terrain-aware CNNs for landslides; diffusion-based nowcasting that leverages frozen LLM blocks; federated personalization with dynamic pruning; and SHAP-guided gradient-boosted models for urban heat. Complementing these methods, the papers introduce practical datasets and tools—site-specific GEDI-derived canopy layers, city-scale DEMs and governance inventories, curated landslide patch datasets, public code for select nowcasting pipelines, and review-driven roadmaps for radar’s AI era. Across these studies, three primary findings emerge: (i) multimodal fusion outperforms unimodal baselines for mapping and hazards; (ii) terrain and structural priors systematically reduce class confusions; and (iii) interpretability and operational constraints (privacy, bandwidth, latency) matter as much as incremental accuracy. Relative to the prior literature, this Special Issue moves beyond narrative review: radar bibliometrics quantify collaboration structure and surface AI-enabled fronts; wetland and species mapping show that structural lidar is now operational in the field of class discrimination; and hazard pipelines deliver governance-ready accounting maps. Practical implications follow directly: for practitioners and agencies, higher-resolution wetland/species maps and governance-ready subsidence accounts sharpen zoning, restoration, and safety interventions; for policymakers, radar bibliometrics illuminate the importance of collaboration hubs and infrastructure priorities while waveform-design principles speak directly to spectrum-sharing policy; and for industry, federated, pruned models and interpretable GBMs offer deployable, privacy-aware analytics for transport and environmental services.

However, gaps persist: the studies included in this Special Issue often cover only a single province or city, leaving small islands and mountain belts underrepresented; uncertainty is seldom propagated end-to-end, cross-site transfer is rare, and segmentation/registration remain manual; physics constraints in deep learning are sporadic; and governance metrics are inconsistent. Outstanding issues include domain shifts across biomes/basins, calibrated end-to-end uncertainty, harmonized benchmarks, scalable physics-informed learning, and open, standardized governance metrics for land deformation. Therefore, a pragmatic agenda follows:

- Build cross-region, multimodal benchmarks with uncertainty (e.g., GEDI+S2+SAR for wetlands/diversity; InSAR+topography for landslides/subsidence) using standard splits and open tools;
- Scale physics-informed fusion by encoding hydrologic/structural priors in losses/architectures and reporting reliability diagrams and per-pixel confidence;
- Run comparative transfer studies that withhold biomes/basins, quantify domain shift vs. added sensors and treat registration quality as a covariate;
- Adopt standards for governance-ready mapping, such as common definitions and reporting;
- Deploy collaboration models that meet operations where they are—through pipelines and preserving privacy, plus reference implementations and good-enough baselines. Together, these papers show that the field has the tools—and increasingly the practices—to turn heterogeneous sensing into decision-grade intelligence; the next step is to standardize how we evaluate, explain, and share it so insights are as portable as the data.

To conclude, we hope that this Special Issue serves as both proof of progress and a platform—a shared starting point for the next generation of trustworthy, scalable sensing technologies that turns complex measurements into better decisions. Last but not least, we extend our sincere thanks to the anonymous reviewers whose thoughtful, constructive, and timely reports materially improved every contribution to this Special Issue. We are grateful to the journal’s Editor-in-Chief, the Associate Editors who handled submissions, and the Managing Editor for their steady guidance throughout solicitation, peer review, and revision. We also thank the production team and publisher staff for careful copyediting and typesetting. Finally, we thank all authors for entrusting us with their work and for their responsiveness throughout the revision process.

